# Nature and Treatment of Diseases of the Ear

**Published:** 1838-01

**Authors:** 


Art. VI.—*The Nature and Treatment of Diseases of the Ear.* By Dr. William Kramer. *Translated from the German, with the latest Improvements of the Author.* By J. R. Bennett, m.d., &c.—*London,* 1837. 8vo. pp. 306.

We gave so full a review of the original of this work (*British and For. Medical Review,* No. V. Jan. 1837,) on its publication, that it is quite unnecessary to say one word, in this place, of its excellence. It is decidedly the best book extant on diseases of the ear, and Dr. Bennett has conferred a great benefit on the profession in this country, by rendering it accessible to every reader. The translation is very well executed. It may be mentioned, as a proof of the great merits of Dr. Kramer's treatise, that it was translated and in preparation for publication, by another gentleman, at the time when the present translation was announced as being in the press. Although we are sorry that our friend was forestalled by Dr. Bennett, the more so as we believe he would have illustrated the original text to a greater extent, by annotations from his own experience, still we are thankful to the author of the present version, which is very creditable to his learning and industry. In these and other signs, we think we see clear indications of a new era in acoustic surgery in this country, when learning and science shall assert their rights, and the despicable ignorance and impudent empiricism which have hitherto prevailed shall be reduced to their proper level.

